# Off-Label Use of Ondansetron in Pregnancy in Western Australia

**DOI:** 10.1155/2013/909860

**Published:** 2013-12-12

**Authors:** Lyn Colvin, Andrew W. Gill, Linda Slack-Smith, Fiona J. Stanley, Carol Bower

**Affiliations:** ^1^Telethon Institute for Child Health Research, Centre for Child Health Research, The University of Western Australia, 100 Roberts Road, Subiaco, WA 6008, Australia; ^2^Centre for Neonatal Research and Education, The University of Western Australia, 374 Bagot Road, Subiaco, WA 6008, Australia; ^3^School of Dentistry, The University of Western Australia, 35 Stirling Highway Crawley, Perth, WA 6009, Australia; ^4^Western Australian Register of Developmental Anomalies, 374 Bagot Road, Subiaco, WA 6008, Australia

## Abstract

*Aims*. Nausea and vomiting of pregnancy is the most common medical condition in pregnancy. There is an increasing trend to prescribe ondansetron although its safety for use in pregnancy has not been established. *Methods*. Exposed pregnancies were all births in Western Australia, 2002–2005, where the mother was dispensed ondansetron under the Australian Pharmaceutical Benefits Scheme, compared with all other births during the same period. Outcomes investigated include maternal and child characteristics, birth defects, pregnancy, and delivery characteristics. *Results*. There were 96,968 births from 2002 to 2005. Ondansetron was dispensed to 251 pregnant women during this period. The women dispensed ondansetron were more likely to be privately insured (OR: 5.8; 95% CI: 4.3–7.9), to be Caucasian (3.3; 1.9–5.7), not to smoke during their pregnancy (2.9; 1.8–4.7), to have a multiple birth (2.7; 1.5–5.0), and to have used fertility treatment (1.8; 1.0–3.4). There was a small but not significantly increased risk of a major birth defect with first trimester exposure (1.2; 0.6–2.2). *Conclusions*. Our study did not detect any adverse outcomes from the use of ondansetron in pregnancy but could not conclude that ondansetron is safe to use in pregnancy.

## 1. Introduction

Nausea and vomiting of pregnancy (NVP) is the most common medical condition in pregnancy, affecting up to 85% of women [1]. The extreme spectrum of NVP is called hyperemesis gravidarum (HG)—affecting 0.5–2.0% of pregnant women [2]. HG is the most common cause of hospitalization in the first half of pregnancy and is second only to preterm labour for pregnancy overall [2–4]. In clinical practice, HG is identified by otherwise unexplained intractable vomiting and dehydration. It is usually associated with weight loss of more than 5% of prepregnancy weight, electrolyte imbalance, and ketonuria [5]. One of the earliest reported sufferers of HG is purported to be the author, Charlotte Brontë, who died from HG in 1855 at the age of 38 years during the fourth month of her first pregnancy [6]. HG was recently reported to be associated with placental dysfunction disorders when it occurs in the second trimester [7]. In Western Australia's main hospital for women, the clinical guidelines for the management of HG with medications include ondansetron as a second line drug therapy, in cases of more refractory vomiting, with failure to improve on first line therapy with antiemetics, pyridoxine, antihistamines and vitamins, and recurrent hospital admissions [1].

Ondansetron is a potent, highly selective 5HT3-receptor antagonist. Its precise mode of action in the control of nausea and vomiting is not known [8]. Ondansetron (tablets, wafers, and injection) is indicated for the prevention and treatment of nausea and vomiting induced by cytotoxic therapy and radiotherapy. Ondansetron (injection) is also indicated for the prevention and treatment of postoperative nausea and vomiting. The product information states that, “as animal studies are not always predictive of human response the use of ondansetron in pregnancy is not recommended [8].” Transplacental transfer during the first trimester of human pregnancies has been reported [9, 10]. The safety of ondansetron for use in human pregnancy has not been established and is assigned pregnancy category B1 in Australia: “Drugs which have been taken by only a limited number of pregnant women and women of childbearing age, without an increase in the frequency of malformation or other direct or indirect harmful effects on the human fetus having been observed. Studies in animals have not shown evidence of an increased occurrence of fetal damage [11].”

Medicines must be entered on the Australian Register of Therapeutic Goods (ARTG) before they can be lawfully supplied in, or exported from, Australia. “Off-label use” prescribing refers to prescribing a registered medicine for a use that is not included or is disclaimed in the product information. Examples include use for a different indication, patient age range, dose, or route to that which is approved by regulatory authorities [12]. Off-label prescribing is not illegal and may sometimes be clinically appropriate, but is associated with a number of clinical, safety, and ethical issues [12]. Most published experience with drugs prescribed during pregnancy for off-label uses has involved either case reports or small subject numbers. There are few well-controlled large studies. In a study of 731 pregnant women conducted in a US nonprivate university clinic, 22.6% took one or more (average 1.7) medicines for off-label indications [13]. A large study in a UK maternity hospital with 17,694 prescriptions in a three-month period found 83% of the medicines prescribed were used off-label with 59% being classified as “caution” or “high risk” for use in pregnancy [14].

The Pharmaceutical Benefits Scheme (PBS) is available to all Australian residents who hold a current Medicare card. It has been in existence since 1948 and is governed by the National Health Act 1953 (Commonwealth) [15]. Under the PBS, the Australian government subsidises the cost of medicine for most medical conditions, with around 80% of prescriptions dispensed in the community, private hospitals and public hospitals (since late 2004) being subsidised. As of December 2005, the scheme covered 804 medicine substances (generic medicines), available in 2,138 forms and strengths (items) and marketed as 3,659 products (brands) [16]. Subsidised medicines are listed in the PBS and most are dispensed by pharmacists for use by patients at home. Medicare Australia compiles information on the supply of medicines through prescriptions subsidised by the PBS.

Ondansetron was first included on the ARTG in April 1991 [17]. Ondansetron is not approved for use in NVP in Australia under the PBS. It is only available as a subsidised medicine under the PBS with an “authority required, management of nausea and vomiting associated with radiotherapy being used to treat malignancy,” or as a “restricted benefit—can only be prescribed for specific therapeutic uses, management of nausea and vomiting associated with cytotoxic chemotherapy being used to treat malignancy which occurs within 48 hours of chemotherapy administration [18].” A recent survey of the management of NVP by obstetricians in Australia found that off-label use of ondansetron in NVP is clearly widespread [19]. In addition to concerns about patient safety, ondansetron is much more expensive than older medications and its use raises the issue of cost both to the patient and the health system in general.

There are relatively few published studies of the safety of ondansetron use in pregnancy. The first reported case was in Greece in 1992 and no birth defects were reported [20]. No birth defects were reported in a single case in the United Kingdom in 1996 [21]. A cohort study from Canadian and Australian teratology information services (*N* = 176 pregnancies exposed to ondansetron) in 2004 reported no significant differences in the frequencies of miscarriage, stillbirth, induced abortion, major malformations, mean birth weight, or mean gestational age [22]. A Swedish record linkage study (*N* = 21 exposed pregnancies) in 2005 did not report any birth defects [23]. A United States case control study (*N* = 55 exposed pregnancies) in 2012 reported an increase in cleft palate defects but not in cleft lip, hypospadias, or neural-tube defects [24]. A case series from Montreal (*N* = 14 exposed pregnancies) in 2012 did not report any birth defects [25]. The largest study was of a Danish cohort published in 2013 (*N* = 1,970 exposed pregnancies). There was no significant increased risk of stillbirth, major birth defect, preterm delivery, or infants born with low birth weight or born small for gestational age [26]. Based on the data available today, ondansetron use cannot be assumed to be safe during pregnancy [27]. In September 2011 the US Food and Drug Administration (FDA) issued a warning about possible serious QT prolongation and torsade de pointes among people receiving ondansetron [28]. The FDA requires strict followup of patients receiving ondansetron to rule out long QT syndromes, electrolyte imbalance, and congestive heart failure or receiving concomitant medications that prolong the QT interval. In the context of NVP, quite a few women with severe NVP might have electrolyte imbalances (hypokalaemia or hypomagnesaemia) [27].

Data linkage of administrative data has been a rich resource for Western Australian (WA) researchers for a number of years [29]. The more recent approval to link national data from the PBS to datasets in the WA Data Linkage System (WADLS) provides new and valuable opportunities to examine birth outcome profiles of prescription medicines dispensed for use during the preconception period and pregnancy. This population-based data linkage study investigated the use of ondansetron in pregnant women giving birth in WA from 2002 to 2005.

## 2. Methods

The exposed pregnancies were all births in WA, 2002–2005, where the mother was dispensed ondansetron under the Australian Pharmaceutical Benefits Scheme (PBS). The comparison group was all other births during the same period. The study used linkable state health administrative data from the WADLS: Hospital Morbidity Data System (HMDS), the Midwives' Notification System (MNS), the Registry of Births and Deaths, and the WA Birth Defects Registry (now called the WA Register of Developmental Anomalies (WARDA)) and the national PBS. The linkages and methodology have been described previously [30, 31]. The WADLS uses the Automatch software package with probabilistic matching based upon medical record number, surname, first given name and initial, date of birth, sex, and address as the principal matching fields. Missed links have been estimated at 0.11% [32]. The WADLS has been validated previously [32, 33] and has been used extensively for health research [29, 34]. The researchers received all data in a deidentified form.

The MNS was introduced in WA in 1974. It is a requirement under the WA Health Act 1911 that a record is to be completed for every baby born, either stillborn or live born, of 400 g or more birth weight and for births at 20 weeks or more gestation occurring in WA. The types of birth attendant recorded in the MNS include obstetrician, other medical officer, midwife, student, self/no attendant, and other.

The WARDA, the first of its kind in Australia, was established in 1980 and records birth defects (BD) occurring in children born on, or after, January 1, 1980 [35]. For the purposes of the WARDA, a BD is defined as a structural or functional abnormality that is present at conception or occurs before the end of pregnancy and is diagnosed by six years of age [36, 37]. The major sources of notification to the WARDA are hospitals and private practitioners, WA Department of Health databases (midwives', mortality, and hospital morbidity systems), and investigative and treatment centres (cytogenetic, pathology, and genetics services). Most minor defects are excluded unless they are disfiguring or require treatment. Of all cases registered, about 90% have at least one major BD (with or without a minor BD); the remainder have only minor defects. A list of exclusions can be found in the annual WARDA report [38]. Each individual defect (up to a maximum of 10 defects per case) is coded according to the 5-digit British Paediatric Association (BPA) ICD-9 system [39]. Syndrome diagnoses are coded along with the major individual defects seen in that infant. The WARDA is a comprehensive source of information on BDs in WA with a high level of ascertainment [37] and is used in relevant areas of health service provision, policy development, research, and evaluation.

Gestational age was estimated using an algorithm taking into account two independent estimates of gestational duration from routinely collected data (last menstrual period (LMP), expected due date, ultrasound fetometry, baby's date of birth, and neonatal estimate of gestational age) by the MNS [40]. The most common procedure during pregnancy in WA in 2005 was uterine ultrasound, with 96.1% of women having undergone this procedure [41]. The variable proportion of optimal birth weight (POBW) [42] was calculated as a measure of the appropriateness of fetal growth. POBW takes into account the major nonpathological determinants of intrauterine growth (IUG). POBW is the ratio of the observed to the “optimal” birth weight; the latter is estimated from a regression equation including terms for gestational duration, maternal height, parity, and infant sex, derived from a total population of singleton births that excluded those exposed to risk factors for IUG restriction, including maternal smoking [43].

The Australian Bureau of Statistics has released Socioeconomic Indexes for Areas (SEIFA) based on the information collected in the five-yearly Census of Population and Housing. These indexes are widely used measures of relative Socioeconomic status at a small geographic area level. The indexes rank and identify areas that are relatively more, or less, disadvantaged. They provide contextual information about the area in which a person lives. The indexes have been obtained by principal components analysis which summarises the information from a variety of social and economic variables, calculating weights that will give the best summary for the underlying variables. The categories of variables include income, education, employment, occupation, and housing [44].

In WA, as soon as practicable after admission to hospital, the patient must elect in writing to be treated as either a public or a private patient. A “private” patient is defined as a person who elects in writing to be treated as an admitted private patient by a medical practitioner of her own choice and is covered for hospital care by a private health insurance fund or is responsible themselves for paying all hospital charges during the admission episode. The patient classification of “public” or “private” is determined from the In-Patients Summary Form (HA22) and recorded in the HMDS [45].

Comparisons were made between women and the children of the women who were dispensed ondansetron during their pregnancy and all other women and children of the women who were not dispensed ondansetron. Odds ratios with 95% confidence intervals (OR; 95% CI) were calculated to assess the strength of association of the maternal and child characteristics. Stepwise logistic regression using the SAS procedure, PROC LOGISTIC, was used to adjust odds ratios (SAS/STAT. Copyright © 2002–2010 by SAS Institute Inc. Cary, NC, USA). For preterm birth and threatened preterm labour, the odds ratios were adjusted for previous preterm birth, smoking during pregnancy, SEIFA, parity, multiple birth, private insurance, and maternal age as these are known potential confounders. Having an obstetrician at the delivery, an elective Caesarean and admission for HG were adjusted for private insurance and multiple birth. The risk of a postpartum haemorrhage ≥500 mLs was adjusted for Caesarean delivery, private insurance, and multiple birth. Independent samples *t*-tests were used to compare means where appropriate. The SAS procedure, NPAR1WAY, was used to compare medians where appropriate.

To fulfil the requirements of our ethics committees' approvals relating to individual privacy, we have not reported cell sizes with less than five study subjects. These are indicated in the results as “<5.” This project has approval from the Human Research Ethics Committees of The University of Western Australia and the WA Department of Health.

## 3. Results

**Figure 1:**
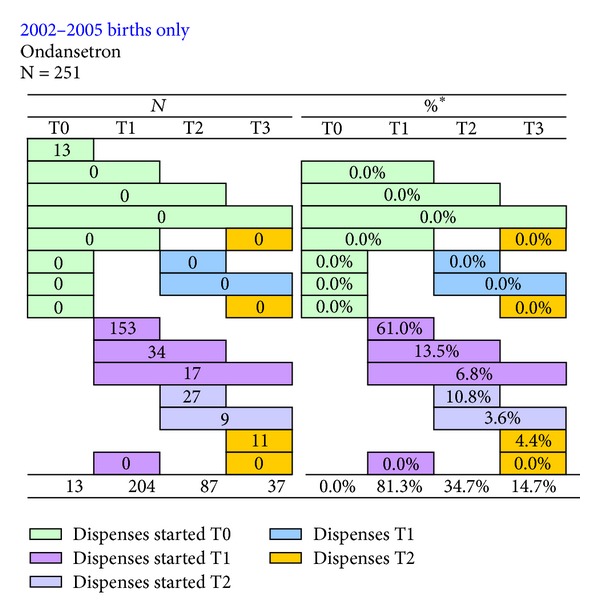
Dispensing patterns of ondansetron from three-months before pregnancy until delivery. *Percentages are the proportion of all birth events dispensed ondansetron within each three-month period.

There were 96,968 pregnancies resulting in a birth in WA from 2002 to 2005. Ondansetron was dispensed to 251 pregnant women (263 children) during this period with an average of 4.7 dispenses per pregnancy. The most commonly dispensed forms were the 4 packs of 4 mg tablet (43%), 4 mg wafer (31%), and 8 mg tablet (21%). All of these were classified in the PBS as a “restricted benefit—management of nausea and vomiting associated with cytotoxic chemotherapy being used to treat malignancy.” There were 2 women dispensed ondansetron and 25 women not dispensed ondansetron with a hospital admission record that included an ICD-10 code relevant to malignancy (C00-C99). The mean (SD) gestational age of the first dispense during pregnancy was 11.9 (6.5) weeks, Figure 1. Ondansetron was the only medicine dispensed to 221 (88%) of the 251 pregnant women that was recorded in the PBS dataset. The most dispenses (36%) occurred during April to June of each year and there were around five times as many dispenses of ondansetron in 2005 as there were in 2002.

The women dispensed ondansetron in pregnancy were more likely to be privately insured (OR: 5.8; 95% CI: 4.3–7.9), to be Caucasian (3.3; 1.9–5.7), not to smoke during their pregnancy (2.9; 1.8–4.7), to have a multiple birth (2.7; 1.5–5.0), and to have used fertility treatment (1.8; 1.0–3.4). Their delivery was more likely to be attended by an obstetrician (adj. 1.7; 1.2–2.4), to be an elective Caesarean (1.3; 1.0–1.8), to be preterm (adj. 1.4; 0.7–2.5) with a median gestation of 38 weeks compared with 39 weeks for other women (*P* < 0.0001), and to be at least the second Caesarean delivery for the mother when compared with other mothers with a previous delivery (1.7; 1.2–2.4), Table 1.

The women dispensed ondansetron in pregnancy were older (mean age: 31.6 versus 29.9 years; *t*(96,696) = 4.71, *P* < 0.0001; median age: 32.1 versus 30.3 years, *χ* ^2^ *P* < 0.0001), taller (mean height: 166.2 versus 164.6 cm; *t*(76,519) = 3.36, *P* = 0.0008), and of a higher Socioeconomic status (mean SEIFA: 1,036.8 versus 997.4; *t*(86,921) = 6.85, *P* < 0.0001) than the pregnant women not dispensed ondansetron.

**Figure 2:**
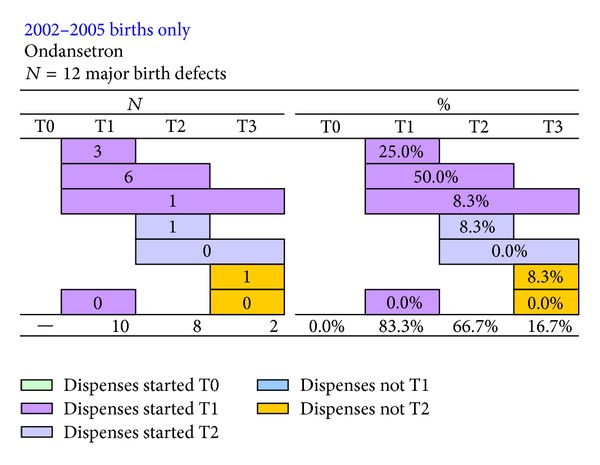
Dispensing patterns of ondansetron from three-months before pregnancy until delivery, to the 12 women with a child with a birth defect.

There was an increased risk of stillbirth (1.8; 0.6–5.5), birth length ≤50 cm (adj. 1.4; 1.0–1.8), birth weight <2500 g (adj. 1.3; 0.8–2.2), and low APGAR score (2.0; 0.6–6.1) amongst children whose mothers were dispensed ondansetron during pregnancy, although none was statistically significant, Table 2. There were fewer than five deaths before the age of one year, in children live born (2.4; 0.8–7.4); all were male, born preterm, and singleton births. There was a 20% nonsignificant increased risk of any major birth defect with first trimester exposure (*N* = 10/211, 4.7% versus *N* = 3,975/98,062, 4.1%): 1.2; 0.6–2.2. There was an increased risk of “753.2 obstructive defects of renal pelvis and ureter” (6.2; 2.0–19.5) but the number of cases was less than five. The dispensing patterns of ondansetron to the 12 women with a child with a birth defect are shown in Figure 2. The women in 10 of the 12 pregnancies were dispensed ondansetron in T1, one in T2 only, and one in T3 only.

Admission to hospital with “excessive vomiting in pregnancy” (ICD-10 O21) was recorded for 35.1% of the women dispensed ondansetron and 1.7% of the women not dispensed ondansetron. The mean (SD) gestational age at first admission to hospital was 10.9 (7.1) weeks for the ondansetron group and 16.7 (11.4) weeks for the other group. Of the women admitted to hospital, 67.0% of the ondansetron group were privately insured compared with 28.4% in the other group.

## 4. Discussion

The women dispensed ondansetron during their pregnancy were more likely to be older, taller, and Caucasian and have an obstetrician at their delivery, have an elective Caesarean delivery, have used fertility treatment, and have a multiple birth and be privately insured and less likely to smoke during pregnancy than those women not dispensed ondansetron. These are all indicators of higher Socioeconomic status and access to health care. We would not usually expect such a greater level of Socioeconomic status and privately insured women represented in the cases for medicines dispensed under the PBS. We have no information on the prescriber or medical practice so we do not know whether the 251 women dispensed ondansetron were under a relatively small group of prescribers or from a broad cross-section of clinicians. This study could only ascertain dispenses of ondansetron to pregnant women with a prescription subsidised under the PBS. It is quite likely that more pregnant women were dispensed ondansetron as a private prescription. The cost of ondansetron under a private prescription during the period of this study was around $44 per pack of 4 tablets and $74 per pack of 8 tablets [46]. It is most likely that these women obtaining ondansetron privately would also be of higher socioeconomic status and visiting an obstetrician specialist and therefore be included in the unexposed group. The result would be to decrease the risks observed towards the null. As the guidelines did not change during the period of the study, the reason for the five times increase in dispensing in pregnancy is unclear.

There was a 20% increased risk of a major birth defect amongst children exposed to ondansetron in the first trimester, but the estimate was based on small numbers and was imprecise, with a wide confidence interval (OR 1.2; CI 0.6–2.2). This is a similar risk to that found by a recent Danish study: OR 1.1; CI, 0.7 to 1.8) [26]. The study was too small to assess risks of individual birth defects although there was an increased risk of “753.2 obstructive defects of renal pelvis and ureter” (6.2; 2.0–19.5). Clarification was sought from WARDA relating to confirmation of obstructive defects of renal pelvis and ureter after birth that may have been detected by ultrasound prenatally. Hydronephrosis that is not present at birth has a different code (753.22) and where the staff cannot find any followup it is recorded as 753.23. All other renal anomalies that do not have a confirmed diagnosis after birth are not registered. In our cohort there were 26 children in the unexposed group recorded with BPA code 753.22 or 753.23 and none in the ondansetron group of children. Excluding these children increased the risk to 7.0; 2.2–22.0.

Preterm birth was more common amongst exposed infants, as were multiple births and a number of other adverse pregnancy outcomes (low APGAR and lower birth weight and length). NVP is more frequently severe in women with multiple pregnancies [2, 47].

Although only a small number of women were dispensed ondansetron in pregnancy in WA, its use increased from 2002 to 2005. The guidelines for the management of hyperemesis gravidarum in WA suggest ondansetron should be used as a second line of therapy. This would follow the use of antiemetics such as prochlorperazine or metoclopramide and then pyridoxine or antihistamines to reduce the nausea. Folic acid and multivitamins are also suggested before the use of ondansetron [1]. Interestingly, the majority of women dispensed ondansetron had not been dispensed any other PBS medication.

In Australia, a working party of the New South Wales Therapeutic Advisory Group was established to address the issue of off-label prescribing of registered medicines and to develop recommendations to guide appropriate practice. One of their recommendations addressed patient consent: “When there is high-quality evidence supporting off-label use of a medicine, the usual process of obtaining consent for treatment should be followed. This includes discussing with the patient/parents/carer the reason for using the medicine, possible alternative therapies and potential side effects. As the medicine is being used off-label, additional information about any uncertainties associated with such use should be given. Documentation of the consent process is recommended and, in some cases, obtaining written consent may be appropriate [12].” With the increasing use of ondansetron for NVP, it is clear that pregnant women should be provided with information relating to the benefits and risks of its use in order to provide informed consent.

## 5. Conclusion

As ondansetron is prescribed off-label in many countries, it is important to accumulate a large cohort of patient outcomes to address its safe use in pregnancy, including investigating the risks for specific birth defects. This study could not conclude that ondansetron is safe to use in pregnancy, given the small but potentially clinically important increases in several measures of outcome investigated. After adjusting for potential confounders, we found an increased risk of a major birth defect (1.2; 0.6–2.2), preterm birth (1.4; 0.7–2.5), shorter birth length (1.4; 1.0–1.8), and maternal urinary tract infection (1.6; 0.9–2.7).

## Figures and Tables

**Table 1:** Maternal characteristics of women dispensed ondansetron any time during pregnancy.

	Ondansetron *N* = 251		Nonondansetron *N* = 96,447		OR (95% CI)
	*N*	%	*N*	%	
Caucasian	238	94.8	81,888	84.9	3.3 (1.9–5.7)
Did not smoke during pregnancy	234	93.2	79,731	82.8	2.9 (1.8–4.7)
Parity > 1	177	70.5	67,267	69.7	1.0 (0.8–1.4)
Multiple birth	11	4.4	1,585	1.6	2.7 (1.5–5.0)
Privately insured	195	77.7	36,012	37.3	5.8 (4.3–7.9)
Not single	245	98.0	87,654	91.3	4.7 (1.9–11.4)
Previous preterm delivery	10	5.6	5,589	8.3	0.7 (0.3–1.3)
SEIFA					
<25%	21	10.0	19,038	24.7	1
25–50%	40	24.5	19,322	28.8	1.9 (1.1–3.2)
50–75%	88	53.3	29,347	37.9	2.7 (1.7–4.4)
>75%	86	36.6	18,981	21.9	4.1 (2.5–6.6)
Labour and delivery characteristics					
Threatened preterm labour, <37 wks*	9	3.6	2,267	2.4	2.3 (1.0–4.9)
Preterm birth, <37 wks*	34	13.5	7,872	8.2	1.4 (0.7–2.5)
Obstetrician at delivery**	183	72.9	39,399	40.9	1.7 (1.2–2.4)
Epidural/caudal anaesthetic	167	66.5	45,272	46.9	2.2 (1.7–2.9)
Caesarean	111	44.2	30,947	32.1	1.7 (1.3–2.2)
Previous Caesarean	52	20.7	13,204	13.7	1.7 (1.2–2.4)
Elective Caesarean**	76	30.3	17,510	18.2	1.3 (1.0–1.8)
Emergency Caesarean	35	13.9	13,438	13.9	1.0 (0.7–1.4)
Postpartum haemorrhage ≥ 500 mls***	18	7.2	8,844	9.2	1.2 (0.7–2.0)
Induced labour	87	34.7	28,475	29.6	1.3 (1.0–1.6)
Threatened abortion, <20 wks	15	6.0	4,964	5.1	1.2 (0.7–2.0)
Analgesia provided	112	44.6	35,289	36.6	1.4 (1.1–1.8)
Artificial rupture of membranes	75	29.9	22,213	23.0	1.4 (1.1–1.9)
Pregnancy characteristics					
Admitted to hospital for HG**	88	35.1	1,646	1.7	32.8 (25.1–42.9)
Fertility treatment	11	4.4	2,340	2.4	1.8 (1.0–3.4)
Urinary tract infection	13	5.2	3,251	3.4	1.6 (0.9–2.7)
Preeclampsia	17	6.8	4,343	4.5	1.5 (0.9–2.5)
Amniocentesis	9	3.6	2,573	2.7	1.4 (0.7–2.6)
Other complications of pregnancy	96	38.2	18,782	19.5	2.6 (2.0–3.3)

*Adjusted for previous preterm birth, maternal age, smoking during pregnancy, SEIFA, parity, private health insurance, and multiple birth.

**Adjusted for private health insurance and multiple birth.

***Adjusted for Caesarean delivery, private health insurance, and multiple birth.

**Table 2:** Characteristics of children of women dispensed ondansetron any time during pregnancy.

	Ondansetron *N* = 263		Nonondansetron *N* = 98,062		OR (95% CI)
	*N*	%	N	%	
Singleton	241	91.6	94,872	96.7	0.4 (0.2–0.6)
Male	124	47.1	50,295	51.3	0.8 (0.7–1.1)
Stillbirths	<5	1.1	635	0.6	1.8 (0.6–5.5)
APGAR 5 mins < 6*	<5	1.1	576	0.6	2.0 (0.6–6.1)
Birth length ≤50 cm**	177	67.3	56,100	57.2	1.4 (1.0–1.8)
Birth weight <2500 g**	30	11.4	7,078	7.2	1.3 (0.8–2.2)
POBW below 1	115	47.7	49,708	52.4	0.8 (0.6–1.1)
Resuscitated at birth	125	47.5	42,376	43.2	1.2 (0.9–1.5)
Any birth defect	16	6.1	4,749	4.8	1.3 (0.8–2.1)
Any major birth defect	12	4.6	3,975	4.1	1.1 (0.6–2.0)
Any major birth defect, first trimester exposure***	10	4.7	3,975	4.1	1.2 (0.6–2.2)

POBW: proportion of optimal birth weight.

*Live births only.

**Adjusted for gestational age, smoking during pregnancy, SEIFA, sex, and parity.

***N = 211 children of a mother dispensed ondansetron in T1.
